# Cross-sectional Census Survey of Patients With Cancer who Received a Pharmacist Consultation in a Pharmacist Led Anti-cancer Clinic

**DOI:** 10.1007/s13187-022-02196-2

**Published:** 2022-07-22

**Authors:** Madeleine Dennis, Aasha Haines, Marie Johnson, Jonathan Soggee, Selina Tong, Richard Parsons, Bruce Sunderland, Petra Czarniak

**Affiliations:** 1 Curtin Medical School, Curtin University, GPO Box U1987, 6845 Perth, Western Australia; 2 Department of Pharmacy, Perth Children’s Hospital, 6009 Perth, Western Australia; 3 Department of Pharmacy, Fiona Stanley Hospital, 6150 Perth, Western Australia

**Keywords:** Oral antineoplastic drugs, Anti-cancer therapy, Patient perceptions, Pharmacist, Oncology clinic, Haematology clinic, Outpatient, Cancer pharmacist, Pharmacist clinic, Cancer centre

## Abstract

Pharmacists have a critical consulting role in patients undergoing oral antineoplastic drug therapy to ensure harm minimisation. Studies exploring the benefits of pharmacists in this role are limited. This study evaluated patient perceptions, experiences and overall satisfaction with clinical pharmacist consultations in patients treated with oral antineoplastic drugs. Data on 160 patients initiated on oral antineoplastic drugs between January 2019 and February 2021 were collected retrospectively from an outpatient Comprehensive Cancer Centre of a quaternary hospital in Western Australia (demographics, cancer type, oral antineoplastic drugs prescribed). In addition, patients were mailed a hard copy questionnaire in March 2021 to assess their satisfaction with pharmacist consultations in the pharmacist clinic, using a 5-point Likert scale. The statements included perceptions of the patient’s understanding, medication adherence, experiences and overall satisfaction with the clinical pharmacist consultation. There were 76 (47.5%) completed questionnaires returned (52.6% female; average age was 63.2 ± 13.9 years). The majority of patients were satisfied with the service offered by the clinical pharmacist (73/76; 96.1%), perceived that clinical pharmacists provided an important service in outpatient cancer care (71/76; 93.4%) and improved their understanding of the use of oral antineoplastic drugs and side-effect management (48/74; 64.9%). Patients’ perceived understanding of their medication regimen and additional health services available improved after pharmacist counselling. The patients also reported overall satisfaction with the service provided by the clinical pharmacist and found it beneficial to their care. The study supports the expanding role of the clinical pharmacist in an outpatient cancer centre.

**Supplementary Information:**

The online version contains supplementary material available at 10.1007/s13187-022-02196-2.

## Introduction

Cancer is a leading cause of death globally and remains one of the leading causes of death in Australia [1, 2]. In 2019, an estimated 144,713 people were diagnosed with cancer in Australia and approximately 49,896 people died [3]. Cancer is characterised as a debilitating, chronic condition which has the potential to impact individuals and their families in multiple ways. In addition to experiencing physical symptoms and psychological distress, there is also a significant economic impact in terms of productivity loss, unemployment and life expectancy [4]. Common cancers, including colorectal, breast, lung, prostate and skin, have increased over the past decade [3].

Traditionally, cancer was managed in hospitals with intravenous (IV) anti-cancer therapies, but advancements in drug technology and the further development of oral antineoplastic drugs (OAD) have made treatment beyond the hospital setting a more viable option [4]. There are many benefits to OAD, including providing patients the freedom to be treated in their own homes, increased independence, closer contact with family and other support structures [5]. Furthermore, oral treatment is less invasive than IV treatment [5, 6].

However, there are barriers to OAD being delivered in the patient’s home. The patients’ lack of understanding about their medications can potentiate inadequate adherence to treatment regimens and suboptimal management of the adverse effects of those therapies. It has been suggested that the potential for ineffectiveness may relate to a lack of expert supervision [7]. Understanding when and how to take the medication can also have a profound effect on medication efficacy and adherence levels. Inadequate compliance can contribute to high variability in patient response and drug exposure, for example, absorption of some OAD can be greatly affected by food intake [8]. Many cancer patients are also taking more than five medications overall and therefore are at greater risk of drug interactions and confusion [9, 10]. A study of 898 OAD patients identified a 46% rate of confirmed drug interactions, with the majority involving the central nervous system [10]. The ability to adequately prevent, correctly identify and manage adverse drug reactions remains a challenge to all health professionals involved in prescribing, dispensing and monitoring OAD [11].

Pharmacists who work in both the community and hospital oncology and haematology settings have specialised knowledge of OAD [9]. The extent to which they are able to expertly communicate this to patients undergoing OAD is important for achieving positive patient outcomes, especially in terms of increased patient understanding of how to manage adverse effects and in increased treatment compliance [12]. Research studies support the inclusion of oncology and haematology outpatient pharmacists in the treatment team as there is a strong correlation between the pharmacist-led counselling of patients and positive adherence to OAD treatment [7, 13, 14, 15]. Research has also shown patients have felt more confident in their drug regimen when counselled by a pharmacist [16]. Despite some evidence being available in other settings, literature investigating the impact of a specialist oncology and haematology pharmacist consultation is lacking in the Western Australian population.

In 2017, a Comprehensive Cancer Centre at a leading quaternary hospital in Western Australia (WA), introduced a Pharmacist Led Anti-Cancer Clinic (PLACC) whereby patients started on an oral anti-cancer medication could be referred for consultation by a clinical pharmacist with training and experience in oncology and haematology. The patients referred to the PLACC were reviewed in an initial clinic, by a clinical pharmacist, where a medication reconciliation was undertaken, to document all prescription and non-prescription (over-the-counter) medications the patient was taking at the time of consultation and to identify potential medication interactions. At the same time, OAD prescriptions were verified against the OAD protocol; dosing adjustments were made where necessary; the patient received medication and side-effect management plan counselling; and supportive care regimens were reviewed. The patients who consented were then booked into a follow-up clinic consultation, with a clinical pharmacist, approximately one week after commencing treatment to assess for early therapy-related adverse effects, compliance and medication access with appropriate escalation of clinical concern.

## Aim

The aim of this pilot study was to evaluate patient perceptions, experiences and overall satisfaction with clinical pharmacist consultations, in patients treated with oral antineoplastic drugs.

## Ethics Approval

The WA Health Governance, Evidence, Knowledge and Outcome system (GEKO) reviewed and approved this study as a Quality Improvement Activity (study number QA39499). Reciprocal ethics approval was also provided by Curtin University Human Research Ethics Committee (study number HRE2021-0026 approved 8th March 2021).

## Method

### Study Design

A retrospective quantitative cross-sectional census survey was conducted at the PLACC at a 783-bed metropolitan quaternary tertiary hospital in WA.

### Participants

Patients initiated on OAD between January 2019 and February 2021, and alive on 11 March 2021, and who were consulted by a pharmacist in the PLACC were included in this study. Patients were excluded if they were receiving concurrent intravenous chemotherapy, were managed exclusively as an inpatient or had no involvement with a pharmacist during their treatment regimen.

### Procedure

Pharmacists kept a record (in the form of an Excel document) of patient referrals to the PLACC, and this record was used to identify potentially eligible patients for the current study. The hospital’s electronic patient medical records database (‘BOSSnet’ and iCM’) was used to determine if these patients met the inclusion criteria for the study (for example, if patients were still alive). The pharmacists’ PLACC notes in the hospitals’ database were used to determine if patients had been initiated on OAD for the first time and had a subsequent interaction with the PLACC. Patient baseline data were collected from a physical review of the hospital database. These included the patient’s date of birth, gender, cancer type, the prescribed OAD agent, whether a medication reconciliation was performed and recorded by the pharmacist, the initial clinic date and whether there was a follow-up consultation with a pharmacist. ‘Medication reconciliation’ was defined as documenting all prescription and non-prescription medications the patient was taking at the time of consultation, to allow for drug interaction checking [17].

Eligible participants were mailed a letter of intent from the hospital explaining the purpose of the study, a hard copy questionnaire and a reply-paid envelope on 11 March 2021. The participants were asked to complete the questionnaire and return it to investigators in the reply-paid envelope by the 12th of April, 2021. Responding to the questionnaire was accepted as consent. A paper-based survey was chosen (in preference to on-line survey) due to the expected age of the study cohort and assumed computer literacy. When an eligible participant was known to have died, they were excluded from the study to reduce the burden on the family.

### Outcome Measures

A novel purpose-designed, retrospective, two-page questionnaire was created by the research team in collaboration with two hospital pharmacists (both of whom were part of the research team) to cover all areas of investigation, including patient understanding, experiences and overall satisfaction. The participants were advised the purpose of the questionnaire was to determine their level of satisfaction with the pharmacist counselling that was undertaken at the hospital during the time they were taking oral anti-cancer medication. The questionnaire consisted of four initial demographic questions (age, gender, level of education, English as a first language) to confirm patient data collected from the hospital database and expand on baseline data. In addition, 16 statements were used to investigate patient satisfaction with the clinical pharmacist. The patients were asked to respond to each statement using a 5-point Likert scale where 1 = strongly agree, 2 = agree, 3 = neutral, 4 = disagree and 5 = strongly disagree. The questionnaire was face and content validated by three specialised oncology and haematology clinical pharmacists, one clinical nurse, one doctor and five cancer patients receiving IV therapy at the 783-bed quaternary hospital.

Each questionnaire was assigned a unique number which corresponded to the patient’s Unit Medical Record Number (UMRN) specific to the Western Australian Health Department. This allowed the returned responses to be linked to the patient’s demographic data. De-identified data were entered into an Excel spread sheet and exported to IBM SPSS version 25.0 (Armonk, NY, USA). Standard descriptive statistics were used to summarise the demographic profile of the patients, and their survey responses. Because some demographic data were available for all eligible subjects, these variables were compared between survey responders and non-responders using chi-square and *t*-tests as appropriate. Seven questions in the survey were designed to assess whether the respondent considered that the pharmacist had helped them to understand the use of OAD, possible adverse effects, and what to do in the case that an adverse effect is experienced. If the responses to all seven questions were either ‘agree’ or ‘strongly agree’ that the pharmacist was able to help, then an overall measure of assistance was classified as ‘provided’. Cross-tabulation of demographic factors against this variable was used to examine whether similar proportions of all subgroups of patients found the assistance helpful. Measures of association between categorical variables were assessed using the chi-square test, with a *p*-value < 0.05 indicating a statistically significant association. Missing values from the questionnaire responses were reported in the study but were not incorporated into any analysis.

## Results

Of 160 eligible patients mailed the questionnaire, 76 completed questionnaires were returned (47.5%). Questionnaire respondents and non-respondents were similar with respect to demographic variables (Table 1), suggesting that the respondents were a fair representation of the pool of eligible participants [respondents: females 40; 52.6%; mean age 63.2 ± 13.9 (range: 29–91) years versus non-respondents: female 44; 52.4%; mean age 58.5 ± 16.9 (range: 18–88) years]. The dominant age bracket was 61 to 80 years (respondents: 47; 61.8% versus non-respondents: 36; 42.8%) with few respondents over 80 years old (6; 7.9%) or under 40 years old (5; 6.6%). Most participants (respondents) spoke English as a first language (61; 80.3%) and had completed up to at least secondary level of education (41/74; 55.4%).

**Table 1:** Questionnaire non-respondent and respondent information (obtained from electronic patient medical record database)

Demographic variable		Questionnaire non-responders		Questionnaire responders		***p*-Value**
		*n*	%	*n*	%	
Mean age, years		58.5 (SD 16.9)		63.2 (SD 13.9)		0.059
Gender	Female	44	52.4	40	52.6	0.975
	Male	40	47.6	36	47.4	
Area	Metro	61	72.6	59	77.6	0.4647
	Rural	23	27.4	17	22.4	
Level of education	Primary	NA		3	4	
	Secondary	NA		38	51.4	
	Tertiary	NA		26	35.1	
	Other	Na		7	9.5	
English is first language	Yes	NA		61	80.3	
	No	NA		13	17.1	
	Missing	NA		2	2.6	
Pharmacist follow up	Yes	48	57.1	50	65.8	0.262
	No	36	42.9	26	34.2	
Referred by	Doctor	83	98.8	73	96.1	
	Nurse	1	1.2	1	1.3	
	Dietician	0	0	1	1.3	
	Pharmacist	0	0	1	1.3	
Five or more medications	Yes	30	35.7	36	47.4	0.313
	No	41	48.8	35	46.0	
	Missing	13	15.5	5	6.6	
Medication reconciliation completed	Yes	52	61.9	57	75	0.076
	No	32	38.1	19	25	
Type of cancer	Colorectal	24	28.6	21	27.6	
	Breast	18	21.4	19	25.0	
	Gastric	5	6.0	5	6.6	
	Melanoma	3	3.6	5	6.6	
	Non-small cell lung	6	7.1	4	5.3	
	Pancreas	4	4.8	3	4.0	
	Gallbladder	2	2.4	2	2.6	
	Myeloma	0	0	2	2.6	
	Neuroendocrine	3	3.6	2	2.6	
	Renal cell carcinoma	3	3.6	2	2.6	
	Other	16	19	11	14.5	

Ten most common cancers identified in questionnaire respondents compared to questionnaire non-respondents are summarised in Table 1. Colorectal cancer was the most common type of cancer in both groups, followed by breast cancer (Table 1). The OAD agent varied dependent on the type of cancer being treated and the most common drug was capecitabine (36; 47.4%). Drugs less commonly prescribed included letrozole (8; 10.5%), palbociclib (7; 9.2%), dabrafenib, everolimus, imatinib and trametinib (*n* = 4; 5.3% for each). Others included exemestane, pazopanib, osimertinin, temozolomide and venetoclax. With respect to medication reconciliation, 25.0% of respondents and 38.1% of non-respondents did not have recorded medication reconciliation.

**Fig. 1:**
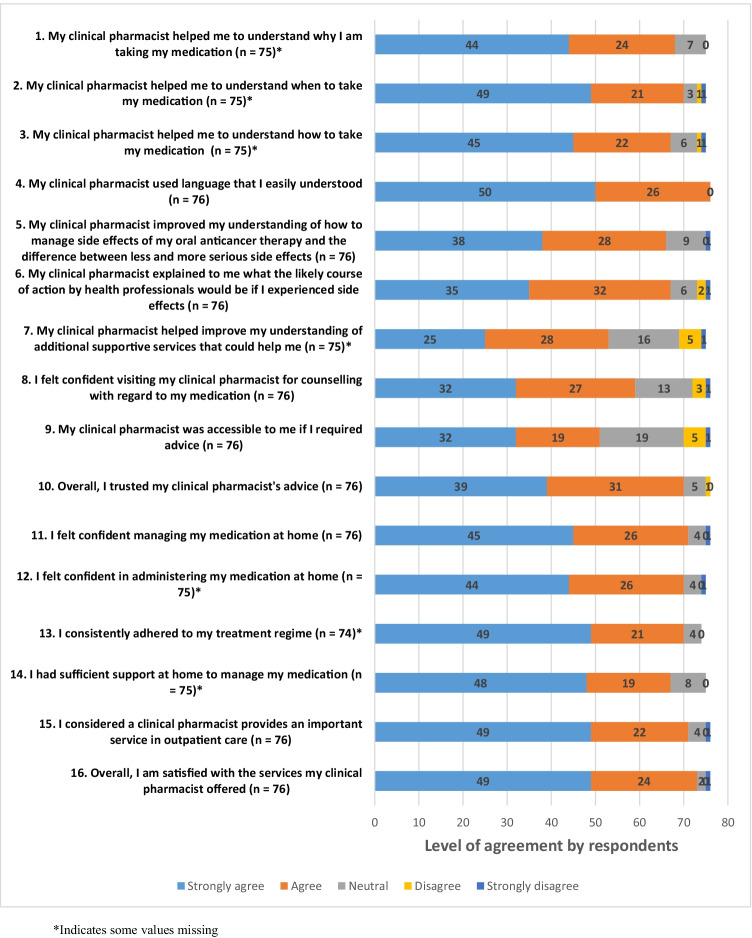
Patient satisfaction with pharmacist intervention. *Indicates some values missing

Questionnaire responses are detailed in Fig. 1. A majority of respondents were satisfied with the pharmacist care and the importance of the pharmacist role in the Cancer Centre, with participants predominantly agreeing with the statements ‘I considered a clinical pharmacist provides an important service in outpatient care’ (71/76; 93.4%) and ‘overall, I am satisfied with the services my clinical pharmacist offered’ (73/76; 96.1%). The majority of respondents agreed that their understanding was improved by the clinical pharmacist counselling on how to manage their adverse effects should they occur, and how to differentiate between less and more serious adverse effects (66; 87.0%).

Of the 74 cases where complete data were available, 48 (64.9%) agreed that the pharmacist had provided assistance (overall) in their understanding of the use of OAD and adverse effects. Cross-tabulating this variable against demographic data showed that no sub-groups stood out as responding differently to other groups. While the proportion of respondents whose first language was English appeared to be more likely to recognise the assistance provided by the pharmacist, compared to those whose first language was not English (68% versus 46%), this difference was not statistically significant (due to the small number of respondents in this latter group: *n* = 13) (Table 2).

**Table 2:** Associations between demographic data and overall assistance provided by pharmacist (summary of questions 8–15)

Variable^a^		Overall assistance provided by pharmacist^b^		*p*-value
		***n*/*N***	**%**	
Gender	Male	22/35	62.9	0.7318**
	Female	26/39	66.7	
Age group	18–40	3/5	60.0	0.7113*
	41–60	11/17	64.7	
	61–80	32/47	68.1	
	81 or more	2/5	40.0	
Area	Metro	36/57	63.2	0.5733**
	Rural	12/17	70.6	
Pharmacist follow-up	No	18/26	69.2	0.5626**
	Yes	30/48	62.5	
Medication reconciliation completed	No	13/18	72.2	0.4523**
	Yes	35/56	62.5	
Five or more medications	No	22/34	64.7	0.8731**
	Yes	22/35	62.9	
English is first language	No	6/13	46.2	0.2002*
	Yes	41/60	68.3	
I felt confident visiting my clinical pharmacist for counselling with regard to my medication (Q8)	Agree	44/58	75.9	0.0002**
	Neutral/disagree	4/16	25.0	
My clinical pharmacist was accessible to me if I required advice (Q9)	Agree	38/50	76.0	0.0038**
	Neutral/disagree	10/24	41.7	
Overall, I trusted my clinical pharmacist’s advice (Q10)	Agree	46/68	67.7	0.1756*
	Neutral/disagree	2/6	33.3	
I felt confident managing my medication at home (Q11)	Agree	48/69	69.9	0.0041*
	Neutral/disagree	0/5	0.0	
I had sufficient support at home to manage my medication (Q14)	Agree	45/65	69.2	0.1131*
	Neutral/disagree	3/8	37.5	
I considered a clinical pharmacist provides an important service in outpatient care (Q15)	Agree	47/69	68.1	0.0486**
	Neutral/disagree	1/5	20.0	

^*^p-Value obtained from Fisher’s exact test

^**^p-Values obtained from the chi-square test

^a^Respondents who answered all the questions 8–16; *n* = 74

^b^The numbers in the column (*n*/*N*) indicate (the number who recognised the assistance provided by the pharmacist) / (total number of respondents who answered the specific question)

Table 2 also shows associations between the ‘assistance provided’ variable and questions 8–16 in the questionnaire, which related to the confidence and trust that the patient had in the pharmacist. Questions where there were fewer than five who responded that they did not agree with these statements were excluded from the table, as these numbers were too small to draw reliable conclusions. In general, those whose pharmacist was accessible and had the trust and confidence of the respondent also tended to agree that they received useful assistance from the pharmacist concerning their OAD medications.

## Discussion

This study explored patient perceptions of a clinical pharmacist consultation in a PLACC. The key finding was that most patients were satisfied with the contribution of the pharmacist and they perceived an improved understanding of their care upon initiation of their OAD.

### Interpretation

Patient satisfaction is often used to evaluate the effectiveness of a service [18]. When a patient is satisfied with the service provided, they are more likely to engage in their care and follow advice provided by healthcare professionals, such as pharmacists [18]. A study conducted in Brazil found that patients rated pharmacists highly in their communication skills and provided valuable information regarding their drug regimen [19]. Similar findings were reported in a study conducted in Maryland, USA, which found the satisfaction rating of the pharmacist role to be overall positive, between 95 and 98% [20]. Pharmacists were evaluated by patients to be delivering comprehensive information that was easily understood and covered key points for their care [19]. The results of this study also presented a high rate of overall satisfaction with the clinical pharmacist (96.1%), which was a statistically significant finding that postulated pharmacist consultation in a PLACC is a valuable asset to an outpatient Comprehensive Cancer Service [20].

Overall, the majority of patients agreed that the pharmacist consultation helped them understand their drug regimen, possible adverse effects they may experience and additional support that was available. Although not investigated in this study, it would be anticipated that patients who have a sound understanding of the pharmacist counselling provided, would show improved adherence to their treatment regimen. Well established evidence in previous studies has shown that where patients have good understanding of their medication regimen, this has a positive impact on compliance [7, 13–15, 20, 21].

The statistically significant results demonstrated that patients who had perceived understanding from the pharmacists counselling, also had the confidence to contact them should they require further assistance or advice. Few participants from rural areas reported they did not have adequate accessibility to a pharmacist. These patients may have relied on other methods of communication with the pharmacist, such as telehealth. Telehealth has become of increasing importance as it allows patients to access a health professional via tele-communicative techniques. Since the start of the coronavirus pandemic in January 2020, telehealth has become a crucial part of the healthcare system [22]. A number of healthcare professionals can provide this service to patients including general practitioners, specialists and allied health providers [22]. One patient who participated in the study lived rurally and did not have access to a pharmacist and therefore would have to contact the Cancer Centre through the telephone should they require assistance. This should be taken into consideration as it outlines the importance of providing pharmacist consultation via telehealth for those patients who do not have ready access to specialised pharmacist consultation.

A majority of patients in this study spoke English as a first language. Not speaking English as a first language could impact understanding of a pharmacists’ counselling advice, which could potentially affect patient’s adherence to their drug regimen. While our data showed a trend supporting this conjecture, it was not statistically significant due to the small numbers involved. For non-English speaking patients, translation services are available at most tertiary hospital facilities. The importance of utilising these services was highlighted in these data and should be a consideration moving forward.

Perceived understanding amongst those who were 60 to 80 years of age trended higher compared to those above 80 years. It is important to note that only a small number of the respondents were aged over 80 (*n* = 5), so it is not possible to draw a conclusion in relation to age. Strategies could be set in place to ensure the best possible outcome with patients who may be at a higher risk of not understanding. For example, allowing the time to speak slowly to the patient and to ensure the information is processed and fully understood.

Patients undergoing anti-cancer pharmacotherapy are at a high risk of experiencing life-threatening adverse effects such as febrile neutropenia, excessive diarrhoea and dehydration and cardiac and thromboembolic events as well as adverse effects such as nausea, fatigue and anorexia [23, 24]. These adverse effects vary with the type of OAD being administered and patient characteristics [23]. Discussion of adverse effects and their management is a routine component of a pharmacy consultation. This is important, as most patients will experience a range of adverse effects when taking OAD, and not all can be addressed and managed in the same way [24]. A literature review suggested that pharmacist counselling in the outpatient cancer centre empowers patients and has a positive impact on their understanding of adverse effects and how to manage them [24]. It was concerning that some respondents and non-respondents did not have recorded medication reconciliation. It was likely more medication reconciliations were carried out without being documented. A reason for this was that during this pilot study, a dedicated full-time pharmacist was not allocated to the PLACC. Therefore, these pharmacists also had other duties to fulfil and though completing the medication reconciliation, may not have had time to immediately record its occurrence, and then overlooked it when they had time.

### Strengths and Limitations

A strength of this study was a sound response rate (47.5%) allowing statistically significant results to be detected. It is rare for questionnaires sent to the public to exceed a 50% response rate ^25^. A second strength was that the investigators who collected, collated and analysed the data, were blind to the patient cohort and had no vested interest in the outcomes of the study.

The main limitations of the study were that data were collected from a single site over a short period of time, such that generalisation to other sites and outpatient pharmacy services should be done with caution. As the study collected retrospective data from January 2019 to February 2021, there was no way of assessing faulty recall. Another limitation that emerged during data analysis was that individual qualitative data were not captured from the participants. A blank space at the bottom of the questionnaire may have been beneficial for patients to add personal comments. This became evident when some participants wrote additional feedback supplementary to the questionnaire statements.

### Further Research

The sample size of this study was relatively small due to the limited number of eligible patients at a single site. Future studies with larger samples from multiple sites may be beneficial to help generalise the findings to wider populations if each site uses a similar methodology. Further research could also expand to qualitative data collection, such as interviews with patients in the months following interactions with the pharmacist. Furthermore, staff perceptions of these services should be a focus of further research.

## Conclusion

The participants strongly supported a pharmacist consultation in a PLACC in a Comprehensive Cancer Centre. The patients reported a high perceived level of understanding and were overall satisfied with the PLACC service. PLACC services were found to have a crucial role in oncology and haematology outpatient care.

## Supplementary Information

Below is the link to the electronic supplementary material.

Supplementary file1 (PDF 149 KB)

## Data Availability

Raw data available on request.
