# Correction to: Cross-sectional Census Survey of Patients With Cancer who Received a Pharmacist Consultation in a Pharmacist Led Anti-cancer Clinic

**DOI:** 10.1007/s13187-022-02203-6

**Published:** 2022-07-30

**Authors:** Madeleine Dennis, Aasha Haines, Marie Johnson, Johnathan Soggee, Selina Tong, Richard Parsons, Bruce Sunderland, Petra Czarniak

**Affiliations:** 1 Curtin Medical School, Curtin University, GPO Box U1987, 6845 Perth, Western Australia; 2 Department of Pharmacy, Perth Children’s Hospital, 6009 Perth, Western Australia; 3 Department of Pharmacy, Fiona Stanley Hospital, 6150 Perth, Western Australia

**Correction to: Biological Trace Element Research**

**https://doi.org/10.1007/s13187-022-02196-2**

The original version of this article unfortunately contained a mistake. The name of “Johnathan Soggee” is now corrected in the author group.

The original article has been corrected.

