# Supplementary material for: Cross-sectional Census Survey of Patients With Cancer who Received a Pharmacist Consultation in a Pharmacist Led Anti-cancer Clinic
Source: J Cancer Educ. 2022 Jul 22;37(5):1553–61. doi: 10.1007/s13187-022-02196-2 (PMC9305046; doi:10.1007/s13187-022-02196-2)
Supplement: Supplementary file 1 — Supplementary file1 (PDF 149 KB) [file 13187_2022_2196_MOESM1_ESM.pdf]

Government of Western Australia
South Metropolitan Health Service

FIONA STANLEY hospital

Curtin University

Oncologist Clinic Patient Questionnaire

Assessment of patient perceptions of a pharmacist led oral anti-neoplastic regime counselling clinic

The purpose of this questionnaire is to determine your satisfaction with the service provided by the Cancer Centre pharmacist who spoke to you about your medications, either face-to-face in a consulting room when you attended the Cancer Centre, or over the telephone. The Cancer Centre Pharmacist spoke to you when you were first prescribed anti-cancer medication by your cancer doctor at Fiona Stanley Hospital.

**Part A: Participant Demographic Details**		
1.	Age: _____ years	
2.	Gender: (please select one)<br>☐ Male<br>☐ Female	☐ Other (specify) ______________________<br>☐ I'd rather not say
3.	Highest Level of Education?<br>☐ Primary Education<br>☐ Secondary Education	☐ Tertiary Education<br>☐ Other, please specify_________________________
4.	Is English your first language?<br>☐ Yes ☐ No	

**Part B: Participant Satisfaction with Pharmacist Intervention**						
Please indicate whether you strongly agree (**SA**), agree (**A**), are neutral (**N**), disagree (**D**) or strongly disagree (**SD**) with each statement by marking an **X** in the appropriate box.						
		**SA**	**A**	**N**	**D**	**SD**
5.	My clinical pharmacist helped me to understand why I am taking my medication	☐	☐	☐	☐	☐
6.	My clinical pharmacist helped me to understand when to take my medication (e.g. morning and/or night, cycle length, treatment length)	☐	☐	☐	☐	☐
7.	My clinical pharmacist helped me to understand how to take my medication (e.g. with respect to food, how many of each tablet, what to do if I have swallowing difficulties)	☐	☐	☐	☐	☐

Government of Western Australia
South Metropolitan Health Service

FIONA STANLEY hospital

Curtin University

Please indicate whether you strongly agree (**SA**), agree (**A**), are neutral (**N**), disagree (**D**) or strongly disagree (**SD**) with each statement by marking an **X** in the appropriate box.

		**SA**	**A**	**N**	**D**	**SD**
8.	My clinical pharmacist used language that I easily understood	☐	☐	☐	☐	☐
9.	My clinical pharmacist improved my understanding of how to manage side effects of my oral anticancer therapy and the difference between less and more serious side effects (e.g. signs of infection, rash, temperature, chest pain, shortness of breath, diarrhoea, nausea and vomiting)	☐	☐	☐	☐	☐
10.	My clinical pharmacist explained to me what the likely course of action by health professionals would be if I experienced side effects (e.g. anti-nausea medication, anti-diarrhoeal medication, dose review)	☐	☐	☐	☐	☐
11.	My clinical pharmacist helped improve my understanding of additional supportive services that could help me	☐	☐	☐	☐	☐
12.	I felt confident visiting my clinical pharmacist for counselling with regard to my medication	☐	☐	☐	☐	☐
13.	My clinical pharmacist was accessible to me if I required advice	☐	☐	☐	☐	☐
14.	Overall, I trusted my clinical pharmacist's advice	☐	☐	☐	☐	☐
15.	I felt confident managing my medication at home after seeing my clinical pharmacist	☐	☐	☐	☐	☐
16.	I felt confident in administering my medication at home	☐	☐	☐	☐	☐
17.	I consistently adhered to my treatment regime	☐	☐	☐	☐	☐
18.	I had sufficient support at home to manage my medication	☐	☐	☐	☐	☐
19.	I consider a clinical pharmacist provides an important service in outpatient care	☐	☐	☐	☐	☐
20.	Overall, I am satisfied with the services my clinical pharmacist offered	☐	☐	☐	☐	☐

Please note that all personal information you provide will be kept confidential and only used for research purposes. Please use the enclosed reply paid envelope to return your completed questionnaire by the **31st of March 2021**.

Thank you for your participation and responses.
